# Validation of pre-operative risk scores of contrast-induced acute kidney injury in a Chinese cohort

**DOI:** 10.1186/s12882-020-1700-8

**Published:** 2020-02-10

**Authors:** Wenjun Yin, Ge Zhou, Lingyun Zhou, Mancang Liu, Yueliang Xie, Jianglin Wang, Shanru Zuo, Kun Liu, Can Hu, Linhua Chen, Huiqin Yang, Xiaocong Zuo

**Affiliations:** 1 Department of Pharmacy, The Third Xiangya Hospital of Central South University, Changsha, China; 2 Center of Clinical Pharmacology, The Third Xiangya Hospital of Central South University, Changsha, China

**Keywords:** Risk scores, Contrast-induced acute kidney injury, Percutaneous coronary intervention, Coronary angiography, Contrast media

## Abstract

**Background:**

Pre-operative risk scores are more valuable than post-procedure risk scores because of lacking effective treatment for contrast-induced acute kidney injury (CI-AKI). A number of pre-operative risk scores have been developed, but due to lack of effective external validation, most of them are also difficult to apply accurately in clinical practice. It is necessary to review and validate the published pre-operative risk scores for CI-AKI.

**Materials and methods:**

We systematically searched PubMed and EMBASE databases for studies of CI-AKI pre-operative risk scores and assessed their calibration and discriminatory in a cohort of 2669 patients undergoing coronary angiography or percutaneous coronary intervention (PCI) from September 2007 to July 2017. The definitions of CI-AKI may affect the validation results, so three definition were included in this study, CI-AKI broad1 was defined as an increase in serum creatinine (Scr) of 44.2 μmol/L or 25%; CI-AKI broad2, an increase in Scr of 44.2 μmol/L or 50%; and CI-AKI-narrow, an increase in Scr of 44.2 μmol/L. The calibration of the model was assessed with the Hosmer-Lemeshow test and the discriminatory capacity was identified by C-statistic.

**Results:**

Of the 8 pre-operative risk scores for CI-AKI identified, 7 were single-center study and only 1 was based on multi-center study. In addition, 7 of the scores were just validated internally and only Chen score was externally validated. In the validation cohort of 2669 patients, the incidence of CI-AKI ranged from 3.0%(Liu) to 16.4%(Chen) for these scores. Furthermore, the incidence of CI-AKI was 6.59% (178) for CI-AKI broad1, 1.44% (39) for CI-AKI broad2, and 0.67% (18) for CI-AKI-narrow. For CI-AKI broads, C-statistics varied from 0.44 to 0.57. For CI-AKI-narrow, the Maioli score had the best discrimination and calibration, what’s more, the C-statistics of Maioli, Chen, Liu and Ghani was ≥0.7.

**Conclusion:**

Most pre-operative risk scores were established based on single-center studies and most of them lacked external validation. For CI-AKI broads, the prediction accuracy of all risk scores was low. The Maioli score had the best discrimination and calibration, when using the CI-AKI-narrow definition.

## Background

Nowadays, iodinated contrast media (CM) have been widely used clinically to improve diagnosis and treatment, with more than 75 million CM used worldwide each year [1, 2]. Acute kidney injury is a common adverse reaction caused by CM. Contrast-induced acute kidney injury (CI-AKI) has become the third prevalent cause of all hospital-acquired renal failure, accounting for 12% [3]. The incidence of CI-AKI was 11% in low-risk population [4], 40% in chronic renal insufficiency population [5] and 50% in diabetic nephropathy population [6]. 18.6% of CI-AKI patients suffered from persistent renal injury, the incidence of chronic kidney disease (CKD) and the total mortality caused by CI-AKI was 7%~ 31%, and the average hospitalization time and social-economic burden increased by 5~10 times [7, 8]. It can be seen that CI-AKI has become an obstacle to the clinical application of CM.

Unfortunately, so far no strategy has been proven to effectively cure CI-AKI [9, 10]. Therefore, the risk scores for CI-AKI are critical to reduce the incidence of CI-AKI. Risk scores can be used to identify high-risk patients who may benefit from preventive strategies such as hydration. Many risk scores for CI-AKI have been established, and the Mehran score, based on percutaneous coronary intervention (PCI) patients in the United States, has been the most classic predictive score and widely used all over the world [11]. However, in our previous study, the accuracy of Mehran score in Chinese patients was limited [12]. Due to population inconsistency, these scores may not be applicable to non-development populations who weren’t included in the derivation cohort.

Many risk scores included operational variables, such as contrast volume, which are usually not known until the procedure is executed. Thus, these scores can only be used after the operation is completed. However, post-operative predictions do not make much sense because only pre-operative prevention measures can reduce the risk of CI-AKI for no treatment strategy. Pre-operative risk scores are more feasible in clinical applications and have been increasingly established. However, most of these pre-operative risk scores lacked effective external validation and are therefore difficult to apply accurately to clinical practice. In this study, our goal was to review and validate the published pre-operative risk scores for CI-AKI and to provide a reference for clinical use of CI-AKI risk scores.

## Methods

### Data sources and searches

We systematically searched the PubMed (1950 to April 2019) and EMBASE (1980 to April 2019) databases for the studies of CI-AKI risk scores. References to all identified articles and previous systematic reviews were also scanned for potential search criteria. The search strategy was provided in detail in Additional file 1: Table S1. Two researchers independently evaluated all design types and screened for all risk scores for predicting CI-AKI. We limited inclusion to studies published in English.

### Study population

A retrospective cohort study was conducted among patients to whom CM was administered for coronary angiography or PCI at the Third Xiangya Hospital of Central South University from October 2007 to July 2017. Nine thousand thirteen patients were identified by the electronic medical record system at the Third Xiangya Hospital of Central South University, Changsha, China. Patients without left ventricular ejection fraction (*n* = 3512), and without baseline Scr and a second Scr within 72 h after procedure (*n* = 2832) were excluded, because without baseline Scr and changed Scr after angiography, CI-AKI could not be determined.

Detailed demographic and clinical characteristics were collected from the structured hospital information system (HIS) including demographics, left ventricular ejection fraction, baseline serum creatinine (Scr) value, high-density lipoprotein, one procedure effected within the past 72 h, urgent PCI, myocardial infarction, diabetes, hypotension, anemia, congestive heart failure, shock, multivessel PCI, previous percutaneous coronary intervention, and acute coronary syndrome. In addition, in order to ensure the accuracy of model verification, all variables were consistent with original studies of the risk scores as much as possible. MDRD formula was used in Chen score and Cockroft and Gault formula was used in Maioli and Lian scores. Thus, in this study the creatinine clearance (CrCl) was calculated by the Cockcroft-Gault (C-G) equation in Maioli score and Lian score: [(140-age) × weight (kg)]/[72 × Scr (mg/dL)] × 0.85 (for female) [13], and the estimated glomerular filtration rate (eGFR) was calculated by the Modification of Diet in Renal Disease equation (MDRD) in Chen score: [186 × Scr (mg/dl)-1.154] × age-0.203 × 0.742 (if female) [14].

### Definition

The primary study end point was the incidence of CI-AKI. At present, the definition of CI-AKI has not been unified, the most commonly used clinical definition comes from the Contrast Media Safety Committee (CMSC) of the European Society of Urogenital Radiology (ESUR), in which renal function has a worsening (Scr increases by more than 25% or 44.2 μmol/L) within 3 days after intravascular administration of CM in the absence of a surrogate cause [15]. However, the relative increase in Scr was found to overestimate CI-AKI with normal renal function, and absolute values were considered to be preferred [16], and many studies used the definition of an increase in Scr of 44.2 μmol/L only, or increase 25 to 50%. The definitions may affect the validation results, so three definition were included in this study: CI-AKI broad1, CI-AKI broad2 and CI-AKI narrow. CI-AKI broad1 was defined as an increase in Scr of 44.2 μmol/L or 25% relative increase in Scr, CI-AKI broad2 was defined as an increase in Scr of 44.2 μmol/L or 50% relative increase in Scr, and CI-AKI-narrow was defined an increase in Scr of 44.2 μmol/L. The earliest Scr concentration within 14 days prior to surgery was defined as baseline Scr, and the highest Scr within 72 h after surgery was used as the follow-up Scr to evaluate the incidence of CI-AKI.

Anemia was defined as a baseline hematocrit value of ≤39% for men and ≤ 36% for women, which were consistent with original studies of Chen score. Hypotension was defined as a systolic blood pressure ≤ 90 mmHg for at least 1 h. Congestive heart failure (CHF) was defined as functional class III or IV of the New York Heart Association. Urgent PCI was defined as the procedure that was implemented within 12 h of admission.

### Statistical analysis

To compare the differences between the different scores, all patients were divided into low-, moderate- and high-risk groups based on the risk scores calculated from patient demographic and clinical characteristics (Table 1). In the studies by Maioli, Chen and Ghani score, patients were divided into four groups: low- risk, moderate- risk, high- risk and very high-risk groups. We classified high-risk and very high-risk groups into the high-risk group in our study. For the Inohara score, the total score ≤ 0, 1 ≤ total scores ≤10, and high total score ≥ 11 were defined as the low-, moderate-, and high-risk groups in this study, respectively.

**Table 1:** Variables in risk scores evaluated

Scores	Variables	Score	Groups		
			Low-risk	Moderate-risk	High-risk
Maioli Score	Age ≥ 73 years	1	score ≤ 3	score 4–6	score ≥ 7
	Diabetes	2			
	Left ventricular ejection fraction≤45%	2			
	Baseline serum creatinine value≥1.5 mg/dL	2			
	Creatinine clearance≤44 mL/min	2			
	Pre-procedure creatinine≥ baseline creatinine	2			
	One procedure effected within the past 72 h	3			
Chen Score	Age ≥ 70 years	4	score ≤ 7	score 8–12	score ≥ 13
	History of myocardial infarction	5			
	Diabetes	4			
	Hypotension	6			
	Left ventricular ejection fraction≤45%	4			
	Anemia	3			
	Estimated glomerular filtration rate < 60(mL/min/1.73 m^2^)	7			
	High-density lipoprotein < 1 mmol/L	3			
	Urgent PCI^a^	3			
Liu Score	Age ≥ 75 years	1	score = 0	score = 1	score ≥ 2
	Left ventricular ejection fraction< 40%	1			
	Serum creatinine> 1.5 mg/dL	2			
Lian Score	Age > 75 years	1.5	score ≤ 1	score 1–3	score ≥ 3
	Creatinine clearance< 60 mL/minute	1			
	Congestive heart failure	1.5			
Lin Score	Age > 75 years	1	score < 1	score 1–2	score ≥ 3
	Hypotension	1			
	Use of intra-aortic balloon pump	1			
	Serum creatinine> 1.5 mg/dL	1			
Ghani Score	Basal creatinine > 115 μmol/L	7	score ≤ 4	score 5–8	score ≥ 9
	Shock	3			
	Female gender	2			
	Multivessel PCI^a^	2			
	Diabetes	2			
Inohara Score	Age		score ≤ 0	score 1–10	score ≥ 11
	≤50	0			
	51–59	1			
	60–69	2			
	70–79	3			
	80–89	4			
	90–99	5			
	Heart failure status	3			
	Diabetes	2			
	Previous percutaneous coronary intervention	−3			
	Hypertension	2			
	Pre-creatinine> 1.0 mg/dL	4			
	Acute coronary syndrome	5			
Zeng Score	Age ≥ 75 years	1	score ≤ 1	score 2–4	score ≥ 5
	Acute myocardial infarction	1			
	Serum creatinine > 1.5 mg/dL	2			
	IABP^b^	2			

^a^*PCI* Percutaneous coronary intervention; ^b^*IABP* intra-aortic balloon pump

IBM SPSS Version 22.0 (SPSS, Inc., Chicago, IL) and R (version2.12.0) were used for all analyses. Continuous variables were expressed as mean and standard deviation (SD). The t-test was used to compare the continuous variables of the normal distribution; otherwise, the Mann-Whiney U-test was performed. The categorical variables were performed by chi-square test. Discrimination and calibration were used to assess score performances. Discrimination is a measure of the ability to distinguish between patients who will and will not develop CI-AKI, as determined by C-statistic, which is tested using the area under the receiver operating characteristic curve [17]. The score was considered to have acceptable discriminating power with a C-statistic > 0.70. Calibration, which measures whether the predicted value of the model is consistent with the probability of occurrence of the final event, as the evaluated by Hosmer-Lemeshow test. All statistical tests were two-tailed, and accepted statistical significance at *P* < 0.05.

## Results

### Overview of risk scores

**Fig. 1:**
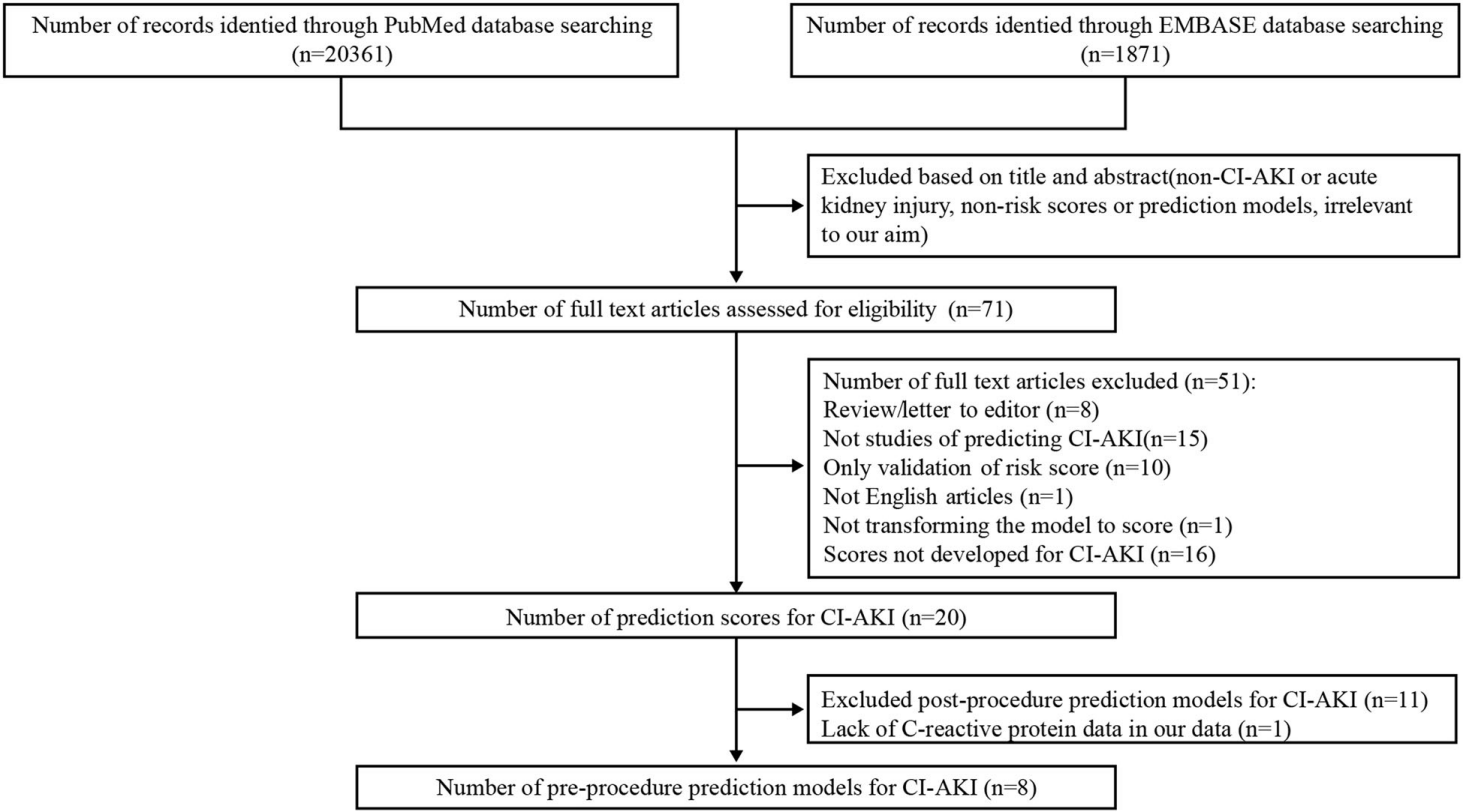
Flow diagram of included studies

Our search strategy yielded 20,361 citations through the PubMed database and 1871 citations through the EMBASE database (Fig. 1). We excluded citations based on screening headlines and abstracts mainly due to non-CI-AKI or acute kidney injury outcomes, non-risk scores or prediction models, animal studies and irrelevant to our goals, leaving 71 full-text articles eligible for evaluation. We subsequently excluded 51 studies with no relevant risk scores for predicting CI-AKI (*n* = 15), reviews and letters to the editor (*n* = 8), only for validating risk scores (*n* = 10), the model in which was not converted to a score (*n* = 1), the scores in which for the risk of CI-AKI was not assessed (*n* = 16), and not an English article (*n* = 1). This produced 20 research risk scores for CI-AKI. In addition, we excluded 11 post-procedure risk scores for CI-AKI according to our goals [18–28], and due to lack of C-reactive protein data, the Athens score was excluded [29]. We ultimately included 8 pre-operative risk scores for CI-AKI in the final validation analysis [30–37].

All studies included patients who underwent coronary angiography or PCI (Table 2), all of whom suffered from diabetes in the Zeng score and the age of patients in the Lian score > 65 years. Only one score (Inohara) was developed in the multi-center. The Inohara score included the largest number of developing patients who were included in the derivation cohort (*n* = 3975) and Ghani had the least (*n* = 247). The incidence of CI-AKI ranged from 3.0%(Liu) to 16.4%(Chen). In addition, the definitions of CI-AKI in the original studies of pre-operative risk scores were defined differently (Table 3), largely because of varying onset time and changes in Scr. Some of pre-operative risk scores defined CI-AKI at 48 h, 72 h, 48–72 h or 5 days of onset, and 5 of them used only an absolute Scr increasing by 44.2 μmol/L but no relative increase. All scores were validated internally by using sliding samples, but only Chen score was validated externally by one cohort from Guangdong General Hospital [38]. Of the 8 scores, 2 had good discrimination in the development cohort and 5 in the validation cohort (C-statistic > 0.8). The most common risk factors for the score included baseline Scr or eGFR or CrCl (eGFR) value (all scores), old age (7 scores), and diabetes (5 scores).

**Table 2:** Characteristics of risk scores development and validation studies for contrast-induced acute kidney injury

Scores	Study population	No. of centers	No. of developing patients	Incidence of CI-AKI (%)	Model Discrimination	No. of validation patients	Incidence of CI-AKI (%) in validation	Model Discrimination in validation
Maioli	patients undergoing coronary angiography or PCI	single	1218	9.4	C-statistic = 0.85	502	10.8	C-statistic = 0.82
Chen	patients undergoing PCI	single	1500	16.4	C-statistic = 0.82	1000	17.2	C-statistic = 0.82
Liu	patients with coronary chronic total occlusion who underwent PCI	single	495	3.0	C-statistic =0.789	233	not reported	C-statistic =0.864
Lian	patients aged > 65 years and who had undergone CAG	single	759	6.3	C-statistic =0.727	527	6.6	C-statistic =0.695
Lin	patients who underwent coronary angiography or PCI if they were diagnosed with STEMI and presented quite high risk in those with NSTE-ACS	single	461	6.9	not reported	231	9.9	C-statistic =0.832
Ghani	patients undergoing PCI	single	247	5.52	not reported	100	5.0	C-statistic =0.61
Inohara	patients undergoing PCI	multi-center	3957	9	not reported	1979	not reported	0.799
Zeng	patients who had been diagnosed with diabetes and underwent CAG/PCI	single	771	3.9	not reported	386	3.9	C-statistic = 0.813

*CI-AKI* Contrast-induced acute kidney injury, *PCI* Percutaneous coronary intervention, *STEMI* ST-segment elevation myocardial infarction, *NSTE-ACS* Non-ST-segment elevation acute coronary syndrome, *CAG* Elective coronary angiography, *AMI* Acute myocardial infarction

**Table 3:** Definitions of contrast-induced acute kidney injury in the original studies of pre-operative risk scores

Scores	CI-AKI definitions
Maioli	an absolute increase in the serum creatinine level by ≥0.5 mg/dL from the baseline within 5 days
Chen	an increase in the serum creatinine level by ≥0.5 mg/dL or ≥ 25% from the baseline within 5 days after PCI
Liu	an absolute increase in the serum creatinine level by ≥0.5 mg/dL from the baseline within 48–72 h after CM exposure
Lian	an increase in the serum creatinine level by > 0.3 mg/dL or ≥ 50% from the baseline within 48–72 h after CM exposure
Lin	an absolute increase in the serum creatinine level by ≥0.5 mg/dL from the baseline within 72 h after CM exposure
Ghani	an increase in the serum creatinine level by ≥0.5 mg/dL from the baseline within 48 h
Inohara	an increase in the serum creatinine level by ≥0.3 mg/dL or ≥ 50% from the baseline after PCI
Zeng	an absolute increase in the serum creatinine level by ≥0.5 mg/dL from the baseline within 48–72 h after CM exposure

*CI-AKI* Contrast-induced acute kidney injury, *PCI* Percutaneous coronary intervention, *CM* Contrast media

### Baseline characteristics and risk of CI-AKI

Of a total of 9013 coronary angiography and PCI patients, 2669 patients were included in the study, and the excluded numbers were showed in Additional file 1: Figure S1. Their demographic, laboratory and procedural characteristics were seen in Table 4 and Additional file 2. Among them, the mean age was 63.31 (±10.21) years, females were 34.75%, and the prevalence of diabetes and hypertension were 39.15 and 54.96%, respectively. The incidence of CI-AKI was 6.59% (178/2699) for CI-AKI broad1, 1.44% (39/2699) for CI-AKI broad2 and 0.67% (18/2699) for CI-AKI-narrow, respectively. Patients with CI-AKI broad1 had a higher prevalence of female, hypotension and anemia. The average age of patients in CI-AKI-narrow cohort was higher. Both eGFR and CrCl were significantly higher in the CI-AKI broads and narrow groups compared to the non-CI-AKI groups.

**Table 4:** Baseline clinical features of the study population

Variable	All patients (*n* = 2669)	CI-AKI_1_ (*n* = 178)	Non-CI-AKI_1_ (*n* = 2491)	CI-AKI_2_ (*n* = 39)	Non-CI-AKI_2_ (*n* = 2630)	CI-AKI_3_ (*n* = 18)	Non-CI-AKI_3_ (*n* = 2651)
Age (years)	63.31 ± 10.21	63.53 ± 9.38	63.29 ± 10.27	64.49 ± 9.35	63.29 ± 10.23	69.38 ± 8.05^*^	63.27 ± 10.21
Female sex	926 (34.75%)	47 (26.40%)^*^	879 (35.29%)	10 (25.64%)	916 (34.83%)	6 (33.33%)	920 (34.70%)
Weight (kg)	64.46 ± 11.25	63.38 ± 10.78	64.54 ± 11.28	63.44 ± 11.85	64.48 ± 11.24	64.84 ± 14.99	64.46 ± 11.22
Diabetes (%)	1045 (39.15%)	68 (38.20%)	977 (39.22%)	17 (43.59%)	1028 (39.08%)	9 (50.00%)	1036 (39.08%)
Hypertension (%)	1467 (54.96%)	97 (54.49%)	1370 (55.00%)	20 (51.28%)	1447 (55.02%)	7 (38.89%)	1460 (55.07%)
Previous PCI (%)	119 (4.46%)	5 (2.81%)	114 (4.58%)	2 (5.13%)	117 (4.45%)	1 (5.56%)	118 (4.45%)
Baseline Scr (μmol/L)	85.93 ± 42.25	75.38 ± 57.39^*^	86.68 ± 40.86	97.92 ± 114.24	85.75 ± 40.26	156.56 ± 149.07^*^	85.45 ± 40.25
Increased Scr (μmol/L)	83.14 ± 41.94	104.11 ± 73.39^*^	81.65 ± 38.31	149.54 ± 140.09^*^	82.16 ± 37.88	229.28 ± 176.18^*^	82.15 ± 37.76
eGFR (mL/min/1.73 m2)	87.24 ± 35.12	113.42 ± 52.27^*^	85.36 ± 32.79	117.78 ± 73.29^*^	86.78 ± 34.06	55.31 ± 26.81^*^	87.45 ± 35.07
CrCl (mL/minute)	74.28 ± 27.01	90.49 ± 37.21^*^	73.12 ± 25.75	93.53 ± 56.38^*^	73.99 ± 26.25	47.33 ± 23.61^*^	74.46 ± 26.94
HDL (mmol/L)	1.17 ± 0.28	1.17 ± .287	1.17 ± 0.28	1.17 ± 0.29	1.17 ± 0.28	1.14 ± 0.29	1.17 ± 0.28
LVEF (%)	56.64 ± 16.95	55.14 ± 17.49	56.75 ± 16.90	55.18 ± 14.94	56.66 ± 16.98	53.06 ± 18.64	56.66 ± 16.93
Myocardial infarction (%)	652 (24.43%)	43 (24.16%)	609 (24.45%)	12 (30.77%)	640 (24.33%)	6 (33.33%)	646 (24.37%)
Hypotension (%)	139 (5.21%)	15 (8.43%)^*^	124 (4.98%)	3 (7.69%)	136 (5.17%)	2 (11.11%)	137 (5.17%)
Anemia (%)	817 (30.61%)	70 (39.33%)^*^	747 (29.99%)	11 (28.21%)	806 (30.65%)	6 (33.33%)	811 (30.59%)
Urgent PCI (%)	13 (0.49%)	0 (0.00%)	13 (0.52%)	0 (0.00%)	13 (0.49%)	0	13 (0.49%)
Shock (%)	20 (0.75%)	1 (0.56%)	19 (0.76%)	1 (2.56%)	19 (0.72%)	1 (5.56%)	19 (0.72%)
CHF (%)	306 (11.46%)	26 (14.61%)	280 (11.24%)	5 (12.82%)	301 (11.44%)	3 (16.67%)	303 (11.43%)
IABP (%)	13 (0.49%)	1 (0.56%)	12 (0.48%)	0 (0.00%)	13 (0.49%)	0	13 (0.49%)
Multivessel PCI (%)	203 (7.60%)	9 (5.06%)	194 (7.79%)	2 (5.13%)	201 (7.64%)	2 (11.11%)	201 (7.58%)
ACS (%)	929 (34.81%)	61 (34.27%)	868 (34.85%)	16 (41.03%)	913 (34.71%)	5 (27.78%)	924 (34.85%)
Previous procedure (%)	231 (8.65%)	16 (8.99%)	215 (8.63%)	2 (5.13%)	229 (8.70%)	0	231 (8.71%)

*CI-AKI* Contrast-induced acute kidney injury, *CI-AKI1* CI-AKI broad1, *CI-AKI2* CI-AKI broad2, *CI-AKI3* CI-AKI narrow, *PCI* Percutaneous coronary intervention, *SCr* Serum creatinine, *eGFR* Estimated glomerular filtration rate, *CrCl* creatinine clearance, *HDL* High-density lipoprotein, *LVEF* Left ventricular ejection function, *CHF* Congestive heart failure, *IABP* Intra-aortic balloon pump, *ACS* Acute coronary syndrome

### Distribution of patients in the different risk categories

**Fig. 2:**
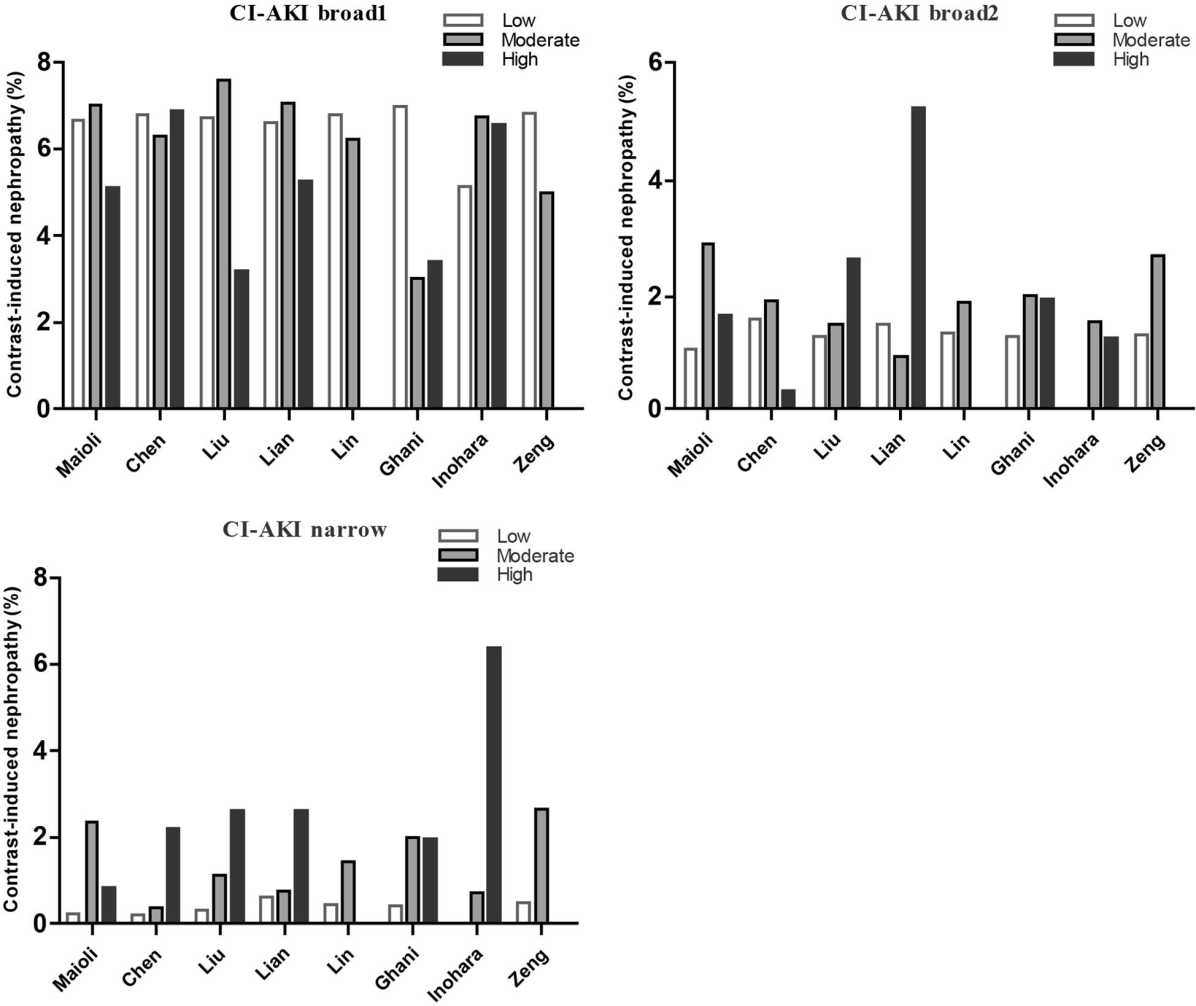
Rates of CI-AKI broad1, CI-AKI broad2, and CI-AKI-narrow in the low-, moderate-, and high-risk groups

All patients were divided into low-, moderate-, and high-risk groups (Fig. 2). When the definition of CI-AKI broad1 was used, the incidence in the low-risk group was not significantly lower than that in the moderate-risk and high-risk groups, and the high-risk group was lower than the moderate-risk group, except for Chen and Ghani. There were no CI-AKI patients in the high-risk group of Lin and Zeng. Lin and Ghani had the highest incidence in low-risk groups. Liu and Lian had the highest incidence in high-risk groups if the broad 2 definition was used, and no CI-AKI patients were found in the high-risk groups of Lin and Zeng and the low-risk group of Inohara. For the Chen, Liu, Lian, and Inohara scores, the incidence of CI-AKI increased with increasing risk when using the CI-AKI-narrow definition.

### Calibration and discrimination

The best calibration was observed for the Maioli score, and the Liu, Lian, Lin, and Inohara scores showed good calibration for CI-AKI, but the Chen and Ghani calibrations express a lack of fit by any definition (*P* < 0.05) (Table 5). For CI-AKI broad1, the AUC for all scores ranged from 0.44 to 0.52, with all risk scores having a low prediction accuracy. For CI-AKI broad2, all risk scores did not show better prediction accuracy, with C-statistics ranging from 0.51 to 0.57. And the scores showed relatively good discrimination. When using the narrow definition of CI-AKI, the C-statistic of Maioli, Chen, Liu, and Ghani were ≥ 0.7, while of Lian and Lin was between 0.5 and 0.6, of Inohara was 0.5.

**Table 5:** Calibration and discriminatory capacity of CI-AKI risk scores

	Maioli	Chen	Liu	Lian	Lin	Ghani	Inohara	Zeng
CI-AKI broad1								
C-statistic (95%CI)	0.49	0.49 (0.45–0.54)	0.49 (0.46–0.52)	0.46 (0.42–0.50)	0.49 (0.46–0.52)	0.44 (0.40–0.48)	0.52 (0.48–0.56)	0.47 (0.43–0.52)
HL (*P)*	0.94	< 0.05	0.67	0.47	0.47	< 0.05	0.35	0.56
CI-AKI broad2								
C-statistic (95%CI)	0.57	0.55 (0.44–0.66)	0.54 (0.46–0.62)	0.51 (0.42–0.59)	0.53 (0.46–0.60)	0.53 (0.44–0.62)	0.55 (0.46–0.64)	0.56 (0.47–0.62)
HL (*P)*	0.91	< 0.05	0.5	0.39	0.51	< 0.05	0.08	0.75
CI-AKI narrow								
C-statistic (95%CI)	0.76	0.76 (0.63–0.89)	0.70 (0.58–0.82)	0.68 (0.57–0.78)	0.63 (0.51–0.75)	0.71 (0.59–0.84)	0.50 (0.36–0.65)	0.63 (0.49–0.78)
HL (*P)*	0.91	< 0.05	0.49	0.35	0.46	< 0.05	0.16	0.75

*CI-AKI* Contrast-induced acute kidney injury, *HL* Hosmer-Lemeshow test

## Discussion

Our study is the first to review the CI-AKI preoperative risk score and perform external validation. In this study, we first systematically evaluated the pre-operative risk scores for CI-AKI. 8 risk scores are only available for patients undergoing coronary angiography or PCI, but not for other procedures such as computed tomography (CT). Only one score was established in a multi-center population, and only Chen score was externally validated. Then we validated these scores externally using the cohort of our hospital. Using the definitions of CI-AKI broad, all C-statistics were less than 0.6, while C-statistic was less than 0.8 using the definition of CI-AKI-narrow. The identification results were widely disadvantageous for CI-AKI. Only three risk scores have a CI-AKI stenosis C-statistic > 0.7, and the Maioli score had the best discrimination and calibration among them.

Many prediction models and scores for CI-AKI have been established, but we only focus on the pre-operative prediction scores in this study because they have better clinical applicability and a series of precautions can be taken to reduce the risk of CI-AKI once identified as a high-risk population. Common interventions include hydration therapy, drug interventions such as alprostadil, discontinuation of nephrotoxic drugs, use of smaller and safer CM, and dialysis treatment [39, 40]. In addition, scores are simpler, more intuitive, and more acceptable to doctors than the original models (such as decision trees and random forests).

Baseline renal function, age and diabetes are common risk factors for pre-operative scores as they have been reported as important risk factors in many prvious studies [16, 40]. Therefore, these risk factors need to attract more attention in establishing predictive scores in the future. For the 8 scores, the Inohara score was developed in the Japan Cardiovascular Database Keio Inter-hospital Cardiovascular Studies (JCD-KICS), a prospective multi-center registry, and the remaining scores were single-center studies, which limit their generalizability. More importantly, seven of risk scores were only validated internally but not externally, so they may not be applicable to other centers due to demographic differences.

The incidence of CI-AKI was extensive in 8 scores studies, the lowest in Liu’s study (3.0%) and the highest in Chen’s study (16.4%). Interestingly, they were all based on the Chinese population, but the incidence was five times different. In fact, the incidence of CI-AKI depends to a large extent on the definition used. Liu defined CI-AKI as an absolute increase in Scr ≥ 0.5 mg/dL over the baseline value within 48–72 h after CM exposure. Chen defined CI-AKI as an increase in Scr from pre-PCI (baseline) level to either ≥25% or ≥ 0.5 mg/dL within 5 days after PCI. Comparing these two definitions, it can be found Liu’s definition is stricter, regardless of the change of Scr or the time of CI-AKI, so the incidence of CI-AKI is lower.

There is no uniform definition of CI-AKI now, as shown in the Table 1, the definitions of CI-AKI in the score studies were quite different. Interestingly, none of the pre-operative risk scores in this study used the definition of CI-AKI published by CMSC. More than half of the models defined the CI-AKI as an absolute elevation in Scr of 0.5 mg/dL when compared with basic Scr. Some studies found the CI-AKI definition of 25% increase in Scr may not be possible in emergency department patients with normal renal function [16, 41]. The definitions can greatly affect the incidence of CI-AKI and the validation results, so in this study we chose 3 definition for a Comprehensive verification.

In our study, all pre-operative risk scores did not show good discrimination when using the CI-AKI broads, but they had better predictive power for CI-AKI narrow. Seven of the scores were validated externally for the first time, and the Chen score was validated externally by one cohort from Guangdong General Hospital [38]. It has good predictive ability (C-statistic =0.828, and 0.746, respectively) with the narrow definition (an increase in Scr ≥0.5 mg/dL) and poor predictive ability (C-statistic =0.555) with broad definition (an increase in Scr ≥25% or ≥ 0.5 mg/dL). Our results were consistent with their results. What’s more, some previous studies have found similar results. We have previously validated the Mehran score and the results suggested that when using the narrow definition (Scr ≥0.5 mg/dL), the Mehran score indicated a good discrimination (C-statistic =0.726), and when using the broad definition (Scr ≥25% or ≥ 0.5 mg/dL), discrimination was limited (C-statistic =0.497) [12]. In a study by Yuan-hui Liu and colleagues, they compared the prognostic value of 6 different risk scores for CI-AKI postoperative scores in 422 consecutive patients with ST-elevation myocardial infarction who underwent primary PCI. These risk scores demonstrated poor discriminatory ability for CI-AKI broad but good for CI-AKI narrow [38].

The CHA2DS2-VASC risk score (CVRS) and the Global Registry for Acute Coronary Events (Grace) were also used to predict CI-AKI. Yong Wang and colleagues found that CVRS, developed for stratification of embolic risk in patients with atrial fibrillation (AF) to provide further optimized anticoagulant therapy, can be used as a simple preoperative predictor of CI-AKI in patients with CTO undergoing PCI (C-statistic =0.742) [42], which was also confirmed in patients with acute ST-elevation myocardial infarction and acute coronary syndrome [43–45]. In addition, the Grace score was also considered to be a strong predictor of CI-AKI development in patients [46, 47].

Further research is needed to develop pre-operative risk scores of contrast-induced acute kidney injury that should use standard definitions to select and measure risk factors in order to reduce misclassification bias and heterogeneity. Reported pre-operative risk scores of contrast-induced acute kidney injury need to be externally validated by multi-center cohorts which can ensure better clinical applicability of risk scores. In addition, Scr threshold for the definition of contrast-induced acute kidney injury is significant to the results of pre-operative risk scores and needs to be accurately defined in the future directions.

### Limitations

There are several limitations in our research explanation that need to be pointed out. First, this is a retrospective single-center study whose inherent weakness are unavoidable. Second, we did not evaluate the end outcomes such as end-stage renal failure and death. Third, we included patients who underwent coronary angiography and PCI, but some scores excluded patients with coronary angiography, some of whom included specific populations, such as the elderly and diabetes; thus, there will be some differences between the characteristics of the development population and the validation population.

## Conclusion

We first performed a review for pre-operative risk scores for CI-AKI, most of which were developed in a single center, lacking external validation, and all of which were focused on patients undergoing coronary angiography or PCI, ignoring other procedures such as contrast enhanced computer tomography (CT). And for the first time, seven of the pre-operative risk score is externally validated, and the validation results are affected by the definition of CI-AKI. Compared with the broad definition of CI-AKI, all pre-operative risk scores have better predictive ability with the definition of CI-AKI-narrow. They expressed poor discriminations for the CI-AKI broads. When using the CI-AKI-narrow, the Maioli score has the best discrimination and calibration, and the 3 scores (the Maioli, Chen, and Ghani scores) have acceptable discriminating power (C-statistic > 0.7).

## Supplementary information

**Additional file 1: Table S1.** Search strategy for contrast-induced acute kidney injury (CI-AKI) risk prediction models. **Figure S1.** Study flow chart.

**Additional file 2: **Data.

## Data Availability

All data generated or analysed during this study are included in this published article and its supplementary information files.

## Abbreviations

AF: Atrial fibrillation
AMI: Acute myocardial infarction
CAG: Elective coronary angiography
C-G: The Cockcroft-Gault equation
CHF: Congestive heart failure
CI-AKI: Contrast-induced acute kidney injury
CrCl: Creatinine clearance
CT: Computed tomography
eGFR: Estimated glomerular filtration rate
MDRD: The Modification of Diet in Renal Disease equation
NSTE-ACS: Non-ST-segment elevation acute coronary syndrome
PCI: Percutaneous coronary intervention
Scr: Serum creatinine
CM: Contrast media
CKD: Chronic kidney disease
SD: Standard deviation
STEMI: ST-segment elevation myocardial infarction
