# Supplementary material for: Validation of pre-operative risk scores of contrast-induced acute kidney injury in a Chinese cohort
Source: BMC Nephrol. 2020 Feb 10;21:45. doi: 10.1186/s12882-020-1700-8 (PMC7011449; doi:10.1186/s12882-020-1700-8)

***Additional files***

**1. Table S1. Search strategy for contrast-induced acute kidney injury (CI-AKI) risk prediction models**

| PubMed and EMBASE Search |  |  |
| --- | --- | --- |
| Medical Subject (MeSH) and EMTREE Headings |  | Title Search Terms |
| “angiography” or “coronary angiography” or “contrast media” or “angiocardiography” or “percutaneous coronary intervention” or “stents or coronary disease” | OR | “coronary angioplast*” or “angiogram*” or “percutaneous coronary intervention*” or “PCI” or “coronary angiograph*” or “cardiac catheterization*” or “coronary revascularization*” or “angiograph*” or “cardiac angiograph*” or “coronary catheterization*” |
| AND |  |  |
| “acute kidney injury” or “creatinine” or “renal insufficiency” | OR | “acute kidney injur*” or “acute kidney failure*” or “acute kidney insufficienc*” or “nephropath*” or “contrast induced nephropath*” or “contrast nephropath*” or “contrast induced acute kidney injur*” or “acute renal failure*” or “acute renal insufficienc*” or “AKI” or “ARF” or “CIN” or “acute renal injur*” or “renal insufficienc*” |
| AND |  |  |
| “risk assessment” or “models, statistical” or “decision support techniques” or “predictive value of tests” | OR | “predict* model” or “predict* rule” or “predict* score” or “prognos* model” or “prognos* rule” or “prognos* score” or “nomogram*” or “decision* rule*” or “risk model*” or “risk stratification*” or “risk algorithm*” or “risk prediction*” or “risk scor*” or “risk prognos*” |

**2. Fig S1. Study flow chart.**


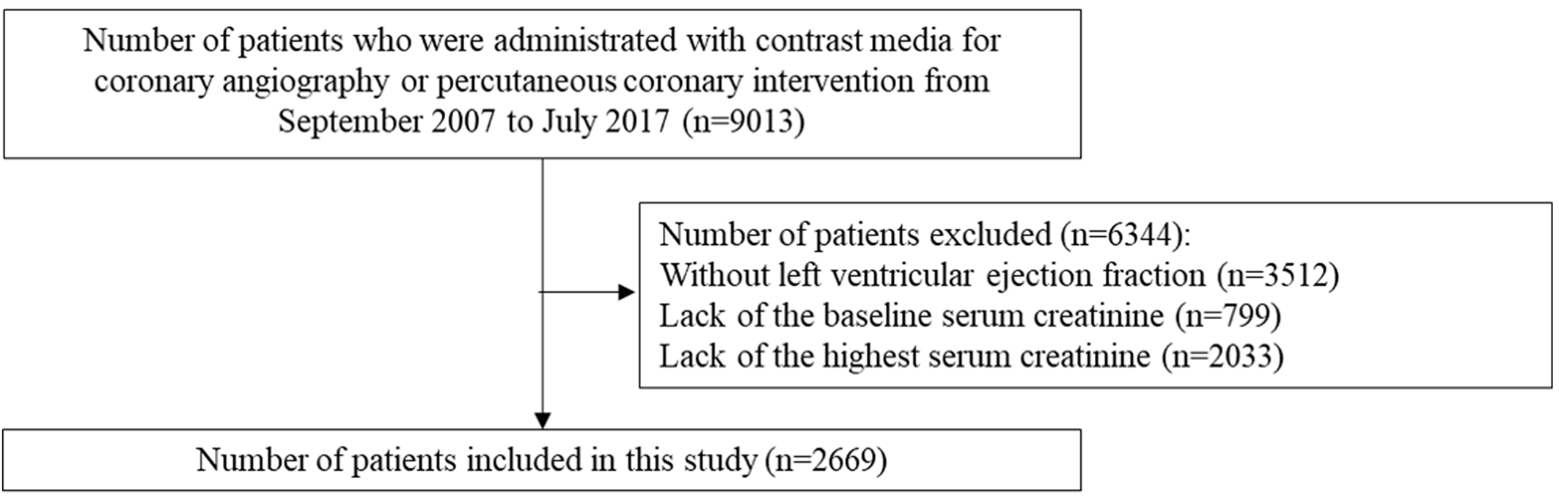

Supplement: Supplementary file 1 — Additional file 1: Table S1. Search strategy for contrast-induced acute kidney injury (CI-AKI) risk prediction models. Figure S1. Study flow chart. [file 12882_2020_1700_MOESM1_ESM.docx]
